# Challenges and opportunities of evaluating work based interprofessional learning: insights from a pediatric interprofessional training ward

**DOI:** 10.3389/fmed.2023.1244681

**Published:** 2023-12-05

**Authors:** Christine Straub, Sebastian F. N. Bode, Jana Willems, Erik Farin-Glattacker, Sebastian Friedrich

**Affiliations:** ^1^Department of General Pediatrics, Adolescent Medicine and Neonatology, Medical Centre, Faculty of Medicine, University of Freiburg, Freiburg, Germany; ^2^Department of Neuropediatrics and Muscle Disorders, Medical Centre, Faculty of Medicine, University of Freiburg, Freiburg, Germany; ^3^Department of Pediatrics and Adolescent Medicine, Ulm University Medical Center, Ulm University, Ulm, Germany; ^4^Section of Health Care Research and Rehabilitation Research, Institute of Medical Biometry and Statistics, Faculty of Medicine and Medical Center, University of Freiburg, Freiburg, Germany

**Keywords:** interprofessional training ward, interprofessional learning, medical education research, work based learning, questionnaire, psychometric properties

## Abstract

**Introduction:**

Interprofessional collaboration among healthcare professionals is fostered through interprofessional education (IPE). Work-based IPE has demonstrated effectiveness within interprofessional training wards. We developed the Interprofessional Training Ward in Pediatrics (IPAPED) and employ a combination of established assessment tools and a newly created IPAPED questionnaire, directed at to assess both students’ learning experiences and program structure. This paper presents the development and analysis of the psychometric properties of the IPAPED questionnaire.

**Methods:**

Nursing trainees and medical students participated in IPAPED. The IPAPED questionnaire was developed to complement established instruments, based on IPE frameworks. Interprofessional collaboration and communication were represented in subscales in part 1 of the questionnaire. Part 2 focused on the IPAPED program itself. Statistical analyses included calculation of internal consistency for part 1 and exploratory factor analyses for part 2.

**Results:**

All IPAPED participants between November 2017 and November 2022 completed the questionnaire (*n* = 105). 94 of 105 questionnaires were analyzed. Internal consistency for part 1 was low (Cronbach’s α <0.58). Exploratory factor analyses revealed three distinct factors: teaching and learning material, interprofessional learning facilitation and professional guidance by nurses on the ward.

**Discussion:**

Our results illustrate the challenge of performing high quality, theory based evaluation in a work-based setting. However, exploratory factor analyses highlighted the opportunity of focusing on both learning facilitators and staff on the wards to ensure a maximum learning output for participants. Developing program-specific questionnaires to gain insight into local structures has the potential to improve work-based IPE formats.

## 1 Introduction

Interprofessional Collaboration (IPC) among healthcare professionals is recognized as a vital strategy to address contemporary healthcare complexities (1). Continuous interprofessional education (IPE) is a crucial prerequisite for equipping learners with the necessary skills, beginning with pre-qualification education and extending through continuing professional development (2). Long-term effects of IPE on later IPC were shown, longitudinal exposure to IPE having an especially positive effect (3). One way of implementing IPE are work based learning formats, such as interprofessional training wards. Compared to seminars, simulations and other, more theory-oriented formats, IP training wards are both particularly challenging to implement and yet rewarding for participants, patients and learning facilitators as they allow for realistic work-placed learning (4). As with many IPE concepts, evaluating effects remains a challenge and there is a need for more data on which concepts do or do not work (5).

In 2017 we implemented the Interprofessional Training Ward in Pediatrics (IPAPED) in a university hospital in south-west Germany (6). From day one of the planning phase, finding and using suitable instruments for evaluation was one of the core ideas behind IPAPED. The concept of frameworks for IPE served as a backbone for both designing the IPAPED concept and deciding on the right kind of evaluation (7). From a range of excellent options, we decided to use the Interprofessional Socialization and Valuing Scale (ISVS) in the 9 item versions and the Interprofessional Collaboration Scale (ICS) (8, 9). While both instruments were validated and widely accepted, we wanted to look at some particularities of our own program in more detail, still bearing in mind the IP frameworks.

Therefore, we developed the IPAPED questionnaire, for both internal evaluation and more insight into effects of the interprofessional intervention. Specifically, we wanted to understand in which way we were able to reproduce theoretical constructs of the program in our findings and how psychometric properties could inform about the continuous improvement process of the program. We wanted to understand whether developing a program-specific questionnaire would be beneficial to the program and could be recommended to teams of other interprofessional training wards as well. After 5 years of running the program and more than 100 students having participated, we evaluated the psychometric properties of the IPAPED questionnaire to answer the following research questions:

1. In which way can we reproduce the theory-based approach in analyzing the psychometric properties?
2. Which factors have an influence on psychometric properties of the questionnaire and how can we address them in the context of the program?
3. How can we use insights from the psychometric testing of the questionnaire to improve the program?

## 2 Methods

### 2.1 The IPAPED program

**Figure 1:**
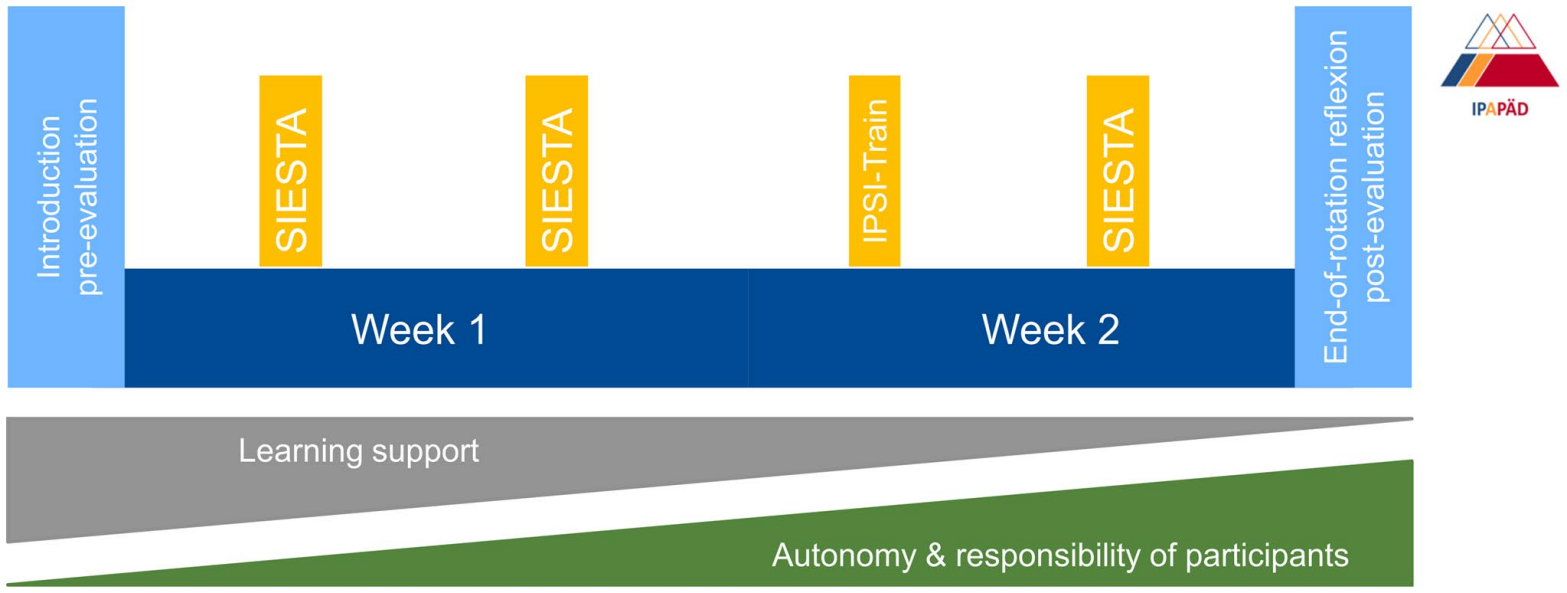
IPAPED – the concept. The two-week rotation is flanked by an introduction session and an end-of-rotation reflection. Pre-and post-evaluations include the ICS, ISVS-9A/B, and the IPAPED questionnaire. Participants need more learning support during the first days of the IPAPED rotation but gain more autonomy and take on more responsibility for patients during the course of the rotation. ICS, interprofessional collaboration scale, IPAPED, Interprofessional Training Ward in Pediatrics, IPSI-train, interprofessional (CPR/resuscitation) simulation, ISVS, interprofessional collaboration and valuing score, SIESTA, speed interprofessional peer teaching pediatric.

The IPAPED program was launched in a general pediatric ward in 2017, welcoming participation from both final-year medical students (MS) and nursing trainees (NT) in their 2nd or 3rd year of training. Interprofessional teams consist of two MS who work 8 AM – 5 PM and four NT who cover morning (6 AM – 2 PM) and afternoon (2 PM – 9 PM) shifts. MS and NT care for 6–8 patients. Nights and weekends are covered by the regular ward team. During the two-week program participants are supervised by registered nurses and board-certified pediatricians as interprofessional learning facilitators who are trained according to an internal curriculum (10, 11). The rotations start with an introduction into interprofessional education, interprofessional collaboration, competencies and roles, handover skills and teamwork. Daily interprofessional handovers and reflections are core elements of the program. Peer teaching elements and an interprofessional resuscitation/CPR simulation-training are included in the 2-weeks course (Figure 1) (12). Parents and patients appreciated the care on IPAPED (13). Participants were very satisfied with supervision, learning success and felt they were able to take on responsibility for patients. They showed an increase of self-perceived interprofessional competencies after the rotation and some positive aspects persisted for up to 1.5 years (5).

### 2.2 Designing the IPAPED questionnaire

The planning phase for the IPAPED program started roughly 18 months before the first run of the interprofessional training ward. Organizational planning that included all relevant stakeholders in medicine and nursing was crucial. During the planning phase, the IPAPED team screened multiple available questionnaires for implementation on the IPAPED. The internationally accepted frameworks for interprofessional education serve as theoretical backbone (7). The Interprofessional Socialization and Valuing Scale (ISVS) in the 9 item versions and the Interprofessional Collaboration Scale (ICS) were selected (8, 9). Participants responded to ISVS-9A/-B and ICS questionnaires at the end of their rotation. These results have been reported previously (6).

Learning facilitators are crucial to successful IPE. Improving our understanding of their role was one aim of the IPAPED program. However, neither ISVS nor ICS contained specific items on the role of learning facilitators and neither do other established instruments. After thorough discussion, the IPAPED team decided against using additional well established instruments, such as the Readiness for interprofessional collaboration scale (RIPLS) or the University of the West of England Interprofessional Questionnaire (UWE IP) (14, 15). As both instruments cover additional aspects of interprofessional learning and collaboration, we decided to develop an additional IPAPED questionnaire, containing elements of both RIPLS and UWE IP. Specifically, RIPLS item 2 and UWE IP item 18 were adapted and specified for items 9 and 10 in the IPAPED questionnaire. IP frameworks and a thorough literature review regarding learning facilitation on interprofessional training wards were taken into account to develop new items for our own instrument. Additionally, the new questionnaire addressed specific aspects of the IPAPED program as well as the learning facilitators.

The IPAPED questionnaire contains 28 items in total. Three are related to sociodemographic data, one is a free-text answer, one relates to IP communication in general and 23 are related specifically to IPAPED. The concepts of interprofessional collaboration and interprofessional communication serve as internal structure (7). Items 5, 6, 7, 8, and 10 were attributed to IP collaboration in the IPAPED context, items 4, 9, 11, and 12 to IP communication on IPAPED. We developed the IPAPED questionnaire in German. For this publication, two members of the research group, one of them being a Native English speaker, translated an English version. The English version of the questionnaire is displayed as Table 1.

**Table 1:** IPAPED questionnaire (English translation).

Item No.	Question	Possible answers
*Sociodemographic information*		
1	Please tell us your profession	Nurse trainee / Medical student
2	Please tell us your gender	Female / male
3	Please tell us your age	…. years
*Please indicate the number that represents your opinion best*		
4	How important do you consider participating in IPAPED during your training?	(1) very important / (2) important / (3) neutral / (4) not important / (5) not at all important
5	How do you rate interprofessional collaboration during daily clinical work on IPAPED?	(1) very good / (2) good / (3) fair / (4) poor / (5) very poor
6	After your rotation on IPAPED, how clear is the understanding you have acquired of your own professional role?	(1) very unclear / (2) unclear / (3) do not know / (4) clear / (5) very clear
7	After your rotation on IPAPED, how do you rate your level of knowledge on the work of the other profession? *Do not rate your own profession*	(1) none / (2) low / (3) sufficient / (4) high / (5) very high
8	After your rotation on IPAPED, how would you rate your motivation to ask the other profession (nurses/doctors) for support regarding patient care in the future?	(1) very high / (2) high / (3) medium / (4) low / (5) very low
9	How much importance would you attribute to interprofessional communication for patient care?	(1) very high importance / (2) high importance / (3) some importance / (4) little importance / (5) very little importance
10	How would you describe the effects of structured interprofessional collaboration during IPAPED on patient care?	(1) very positive effects / (2) positive effects / (3) neither positive nor negative / (4) negative effects / (5) very negative effects
11	Giving and receiving feedback is a core element of IPAPED. How satisfied are you with the feedback culture during your rotation on IPAPED?	(1) very satisfied / (2) satisfied / (3) indifferent / (4) unsatisfied / (5) very unsatisfied
12	What suggestions would you provide the organizers about running the IPAPED course in future?	Keep the program without changes / keep the program with changes / abolish the program / do not know
13	If you marked „keep the program but change it“, what would you change?	*Free text answer*
*Please rate your satisfaction during your rotation on IPAPED regarding…*		
14	…guidance of the interprofessional collaboration by nurse learning facilitators.	(1) very good / (2) good / (3) fair / (4) poor / (5) very poor
15	…guidance of the interprofessional collaboration by physician learning facilitators.	
16	…professional guidance and support from the nursing staff on the ward.	
17	…professional guidance and support from the doctors on the ward.	
18	…the IPSI emergency training	
19	…the introductory event	
20	…the interprofessional midday reflection	
21	…the SIESTA teaching session	
22	…the learning objectives	
IP 22	…the selected medical conditions	
24	…the feedback rules	
25	…the IPAPED pocket guide	
26	…the learning diary	
27	…the teaching and information materials as a whole	
28	Please provide an overall grade for IPAPED	(1) very good / (2) good / (3) fair / (4) poor / (5) very poor

### 2.3 Data collection

Data were collected during IPAPED rotations between November 2017 and November 2022. During that time, 44 MS and 61 NT participated in IPAPED. The IPAPED rotations took place on three wards, with two of them in the same hospital. The 105 participants all completed the paper-based questionnaire at the end of their rotation. The answers were transferred into an electronic spreadsheet by an independent member of the IPAPED team who was not involved in the analyses.

### 2.4 Statistical analysis

Based on the theoretical considerations, we tested the questionnaire for internal consistency (Cronbach’s α and McDonald’s ω). As a second step we performed exploratory factor analyses (EFA; extraction method: principal component analysis after varimax rotation) to allow for the identification of new scales and concepts. We decided to only include items 14 to 28 for the EFA because of their consistent Likert scale. Items 4 to 12 had Likert scales, but with varying labels, making them difficult to include for further analysis. Item 13 was omitted because it was a free text answer. Kaiser-Mayer-Olkin (KMO) coefficient and Bartlett sphericity test were used to analyze the suitability of the data for EFA. Items for EFA were first screened in a missing value analysis, excluding items with more than 20% missing values. After a first EFA, items 19, 21, and 24 were removed because of double loading on two separate factors. Because the first EFA was mainly conducted to provide support for selecting items, only the second EFA’s results are reported in detail below. We performed factor analyses in IBM SPSS® version 29.0.0.

## 3 Results

### 3.1 Sample characteristics

There was a 100% return rate of the surveys (*n* = 105), with 58% (*n* = 61) NT responses and 42% (*n* = 44) from MS. The majority of the participants were female (*n* = 93; 88.5%), with all but one of the male cohort being MS. The mean age was 22.2 years for NT and 26.8 years for MS. Full sample characteristics can be found in Supplementary Table S1.

### 3.2 Psychometric properties

Items 5, 18, and 26 were excluded from the analysis due to a high rate of missing values (>20%). Items 4–12 were separated into two different subscales, based on frameworks of interprofessional education (7). Because items 14–28 were tailored to meet IPAPED specific aspects they were not included in the first analyses. The first theory-based subscale referred to the concept of “interprofessional collaboration,” comprising items 4, 5, 6, 7, 8, and 10. The second subscale referred to the concept of “interprofessional communication,” comprising items 9, 11, and 12. For both subscales, internal consistency was low with Cronbach’s alpha α = −0.378 for subscale 1 and α = 0.505 for subscale two (see Table 2). We were thus not able to reproduce the theory-based background of the questionnaire by testing for internal consistency of the subscales. Potential factors relating to this finding are addressed in detail in the discussion section of the manuscript.

**Table 2:** Values for internal consistency of theory-based subscales.

Subscale	Interprofessional collaboration	Interprofessional communication
Items	5, 6, 7, 8, 10	4, 9, 11, 12
Cronbach’s α	−0.378	0.505
McDonald’s ω	Not available	0.552

The second part of the questionnaire consisted of items 14–27. From the original dataset of 105, only 94 were explored for EFA because of missing data. The KMO coefficient (KMO = 0.700) and Bartlett sphericity test (χ2 = 217.74, *p* < 0.001) indicated that data were suitable for exploratory factor analysis. As described in the methods section, items 19, 21, and 24 were removed from the analysis. After removal of these items, Kaiser-Guttman criterion suggested a three-factor solution. The three factors explained 58% of the variance (factor 1: 32%, factor 2: 15%, factor 3: 11%). The items’ standardized loadings were ≥ 0.60 on factor 1 (items 20, 23, 25, 27, and 28); ≥ 0.64 on factor 2 (items 14, 15, and 17) and ≥ 0.69 on factor 3 (items 16 and 22). Factor 1 showed sufficient reliability (Cronbach’s α = 0.725, McDonald’s ω = 0.731). Factor 2 had limited reliability (Cronbach’s α = 0.571), with McDonald’s ω not reportable (only 3 items). Factor 3 consisted of two items only, thus no internal consistency testing was possible. Loadings for all factors and reliability measures can be found in Table 3.

**Table 3:** Loadings for all factors 1–3 of exploratory factor analysis, including values for internal consistency.

	Factor 1	Factor 2	Factor 3
Item 25	0.746		−0.218
Item 28	0.659	0.342	0.313
Item 20	0.651	0.105	
Item 23	0.624		0.503
Item 27	0.600	0.128	0.160
Item 15		0.798	.-127
Item 17	0.173	0.740	0.130
Item 14		0.638	0.227
Item 16	−0.107	0.337	0.761
Item 22	0.436		0.692
Cronbach’s α	0.752	0.57	-
McDonald’s ω	0.731	-	-

The final step was evaluating the factor content. Factor 1 items focused mainly on teaching and learning material, medical conditions and overall appreciation of the IPAPED program, so it was named “teaching and learning material.” Factor 2 was named “interprofessional learning facilitation” because it contained items focused on the interprofessional nurse and physician facilitation and guidance by ward physicians. Factor 3 “professional learning” consisted of the professional guidance by nurses on the ward and learning objectives.

## 4 Discussion

In this study, we describe the development, implementation and analysis of psychometric properties of a questionnaire designed for evaluation of the interprofessional training ward in pediatrics, named the IPAPED questionnaire. Areas of evaluation comprise self-reported aspects on interprofessional communication and collaboration as well as feedback on aspects specific to the program itself, including interprofessional learning facilitators.

The IPAPED questionnaire was designed as a complementary tool for our interprofessional training ward, with the main focus of evaluation of the program itself. Our short, complementary survey focused on interprofessional collaboration, communication and learning frameworks, covering aspects missing from the ISVS-9A/-B and ICS questionnaires. We did not identify any other established instrument that would have covered all aspects of the IPAPED that we deemed important, especially learning facilitation.

However, we were unable able to reproduce the theory-based background of the questionnaire by testing for internal consistency of the subscales. There are several possible reasons for this challenge: Firstly, only the first section of the questionnaire (items 4 to 12) related specifically to the concepts of interprofessional collaboration and communication (7). The numbers of items for both concepts (IP collaboration: five, IP communication: four) are comparable to other established questionnaires. The ICS, for example, contains three subscales with 5, 5, and 3 items each (9). One major problem with items 4–12 of our questionnaire might be the inconsistent labeling of the Likert type answer scales. To achieve consistent answers and facilitate analysis of psychometric properties, questions should be re-phrased in a way to allow for one same Likert scale for all items. Feasibility of this approach in an IP context has been elegantly demonstrated by the ISVS and the individual Teamwork Observation and Feedback Tool (iTOFT) (8, 16).

One other challenge is presented by the fact that answers were collected over a relatively long period after rotations with 4 to 8 students each. Answers might have been influenced more by the individual experience related to the particular group than by the program itself. Emotions, both positive and negative have an important impact on IP learning experiences (17). These limitations are related to the work-based nature of the program, which prompted constant small changes in the program and a relatively small number of students per rotation. However, the work-placed learning and the living program with constant changes are suggested by participants, patients, and faculty, as major strengths of the IPAPED program.

The second half of the questionnaire was directed at more specific aspects of the IPAPED, such as learning aids and learning facilitation. Exploratory factor analyses revealed different opportunities:

Factor 1, “teaching and learning material,” had the strongest influence on overall rating and variance. This is consistent with findings by other groups that emphasize the importance of a clear structure in the changing context of work based interprofessional education (18). In our case, this included structured concepts for ward rounds on pocket cards and a selection of patients with clearly defined medical conditions in order to leave more space for interprofessional aspects of learning (6, 10). Notably, the daily team reflection at lunchtime is part of this most important factor. These 30 min were dedicated at reviewing on the past 24 h, giving space for urgent problems and enabling the team to adjust the learning goals and learning process. Learning facilitators encouraged a culture of speaking up and listening, creating a “safe place with space for learning” (19).

Factor 2, “interprofessional learning facilitation,” summarized ratings for interprofessional learning facilitators, both nurses and physicians and guidance by physicians on the ward. The latter gave profession-specific instructions and medical advice to the team. Faculty development for interprofessional education in general and work based formats in particular has recently been a field of increasing interest (20, 21). Among IPE experts, there is a consensus that high-quality IPE needs effective faculty training, comprising reflection on roles and responsibilities, team communication and professional identity (22). One of core roles of physicians as defined in the CanMEDs concept is being a “member of a team” (23).

Factor 3 yielded the most surprising results, distinguishing the item “…professional guidance and support from the nursing staff on the ward” alongside the “learning objectives” from the other items mentioned above. There are several possible explanations for this finding, some of which might be transferrable to other wards and contexts: Ward nursing teams tend to be more permanent and stable than residents or other junior doctors, who frequently change. For example, on the three wards where IPAPED took place, two residents worked on the ward for a period of 6 months, whereas some of the nursing staff had more than 30 years of experience and had been part of the same ward team for several years. The nursing teams might thus be considered small examples of communities of practice (24).

Introducing a change process, such as an interprofessional training ward, can be challenging. This holds particularly true when teams work in shifts and it is never possible to have all members of a team present at meetings, workshops etc. Establishing the structures needed for a sustainably successful IP training ward involves convincing important stakeholders as well as the colleagues affected by the teaching format (1). The possible explanations given so far focus on the nursing teams “being different from the rest.” From a students’ perspective it could also be a sign of appreciation: Guidance from the nursing staff on the ward was associated with the formal learning objectives. These included both profession-specific, as well as interprofessional items. The questionnaire does not distinguish between those two groups. Yet, informal learning from nurses has been reported for junior doctors, with implications for interprofessional education (25). Findings from our EFA should encourage faculty development including nursing teams of interprofessional training wards. Making this resource available to learners can be crucial and having the nursing team on board is essential to ensuring a successful program in the long-term.

Strengths of our study include the work-based nature, since evidence on real life IP is still scarce. One excellent example was able to demonstrate optimized antimicrobial treatment, improved quality of care and economic outcome (26). In addition, the continuous implementation over 5 years can be considered beneficial, since data come from a well-established program that is still ongoing and can be used for further iterations. Lastly, our high response rate of 100% was possibly due to small groups, with personal contact to each student and questionnaires kept to a minimum in length.

Limitations include a relatively long time of data acquisition, which naturally led to constant changes within the relevant wards (e.g., physician teams). Also, despite a structured training program, there were frequent changes in learning facilitators (5 nurse learning facilitators and 4 physician learning facilitators in total) (21). Even though *N* = 105 is a considerably high number of participants for work based IP programs, it is still relatively small for robust statistical analyses. The reported statistical results should therefore be considered with caution and provide more of an exploratory framework regarding a potential structure of the questionnaire. Items 4 to 12 had Likert scales, but with varying labels. This can be a challenge when discussing further analysis.

Values for internal consistency were rather low even for data driven EFA. Further statistical tests, such as fitting the data to classical-test-theory-based models (CFA), could not be performed. Item 21 on “feedback” was removed from the analysis at the very beginning, because of double loading on two factors. Feedback is considered a crucial element of IP learning formats and collaborative practice (1). However, our item did not distinguish between feedback among students (peer feedback), feedback from learning faculty and the overall feedback culture, e.g., between learning facilitators and the staff on the ward. Future studies could explore this aspect more closely.

Future improvements of the questionnaire should also aim at identifying additional items, based on existing concepts, such as IP frameworks, or by using qualitative methods such as focus groups (7).

In conclusion, our analysis of the psychometric properties of the IPAPED questionnaire did not allow us to replicate theory-based subscales in the first section of the questionnaire. Nevertheless, these aspects were already well addressed by established instruments like ISVS and ICS. The attempt to provide additional granularity through a supplementary questionnaire encountered challenges. For specific aspects of our program, however, the data driven analysis yielded interesting results. Establishing short, program-specific instruments with analysis of psychometric properties could therefore be useful to identify areas of improvement on interprofessional training wards.

## Data availability statement

The raw data supporting the conclusions of this article will be made available by the authors, without undue reservation.

## Ethics statement

The studies involving humans were approved by University of Freiburg ethics committee, permit no. 561/17. The studies were conducted in accordance with the local legislation and institutional requirements. The participants provided their written informed consent to participate in this study.

## Author contributions

CS and SB designed and implemented the IPAPED program. CS designed the IPAPED questionnaire. SF prepared the data for analysis. JW and EF-G analysed the data. SB and SF wrote the manuscript and prepared the figures. All authors revised the manuscript and agreed on the final and revised version.
