# Supplementary material for: Challenges and opportunities of evaluating work based interprofessional learning: insights from a pediatric interprofessional training ward
Source: Front Med (Lausanne). 2023 Dec 5;10:1244681. doi: 10.3389/fmed.2023.1244681 (PMC10729382; doi:10.3389/fmed.2023.1244681)
Supplement: Supplementary file 1 [file Table_1.docx]

# Supplementary Material

Supplementary Material includes supplementary table 1.

Supplementary table 1: Sample descriptive characteristics

| Profession | Nurse trainees n=61 (58%) | Medical students n=44 (42%) |
| --- | --- | --- |
| Gender | Female n=93 (88.5%) | Male n=12 (11.5%) |
| Gender/Profession | Nurse trainees: female n=61 (100%) | Medical students: female n=32 (73%), male n=12 (27%) |
| Age/Profession (mean, SD) | Nurse trainees 22.2y (SD=2.1) | Medical students 26.8y (SD=2.4) |
